# Exosomes and pancreatic diseases: status, challenges, and hopes

**DOI:** 10.7150/ijbs.35823

**Published:** 2019-07-20

**Authors:** Xiao-Yu Guo, Fan Xiao, Jie Li, Yi-Nan Zhou, Wang-Jun Zhang, Bei Sun, Gang Wang

**Affiliations:** 1 Department of Pancreatic and Biliary Surgery, The First Affiliated Hospital of Harbin Medical University, Harbin, Heilongjiang Province, China.; 2 Department of Gastroenterology, The First Affiliated Hospital of Harbin Medical University, Harbin, Heilongjiang Province, China.

**Keywords:** Exosomes, Pancreatic cancer, Acute pancreatitis, Chronic pancreatitis, Noncoding RNA

## Abstract

Pancreatic disease, including pathologies such as acute pancreatitis (AP), chronic pancreatitis (CP), and pancreatic cancer (PC), is a complicated and dangerous clinical condition involving the disruption of exocrine or endocrine function. PC has one of the highest mortality rates among cancers due to insufficient diagnosis in early stages. Furthermore, efficient treatment options for the disease etiologies of AP and CP are lacking. Thus, the identification of new therapeutic targets and reliable biomarkers is required. As essential couriers in intercellular communication, exosomes have recently been confirmed to play an important role in pancreatic disease, but the specific underlying mechanisms are unknown. Herein, we summarize the current knowledge of exosomes in pancreatic disease with respect to diagnosis, molecular mechanisms, and treatment, proposing new ideas for the study of pancreatic disease.

## 1. Introduction

Pancreatic diseases, which primarily include acute pancreatitis (AP), chronic pancreatitis (CP), diabetes mellitus, and pancreatic cancer (PC), occur in >10% of the world population and are potentially related to the disruption of exocrine and endocrine function.^1^ AP is a common inflammatory disorder of the pancreas accompanied by potentially severe local or systemic complications and high mortality.^2^ CP encompasses a wide spectrum of fibroinflammatory disorders of the exocrine pancreas, and currently, the primary therapy for CP comprises symptomatic treatment.^3^, ^4^ PC is a highly lethal disease with extremely poor prognosis, and the 5-year survival rate of PC patients remains as low as 6%.^5^ Because of the high recurrence rate and low initial resection rate, the survival time prolongation in PC is still unsatisfactory.^6^ Furthermore, pancreatic diseases can undergo transformation. For example, recurrent AP has a high risk of conversion to CP, and CP accompanied by pancreatic fibrosis may eventually become PC.^4^, ^7^ In general, pancreatic diseases continue to confound clinicians and researchers, particularly with respect to the pathogenesis of AP, definitive diagnosis of early stage PC, and discovery of effective therapies aimed at mechanisms of disease pathogenesis, all of which require additional extensive, in-depth studies.

In recent decades, exosomes have become a hot topic for researchers and clinicians worldwide. As a type of extracellular vesicle, exosomes can be secreted endogenously from nearly all cell types and exist in various bodily fluids, such as blood, saliva, breast milk, cerebrospinal fluid, amniotic fluid, bile, seminal fluid, ascites, feces, and bronchoalveolar lavage fluid.^8^, ^9^ Initial studies regarded exosomes as merely 'excretory vesicles' for removing the transferrin receptor during reticulocyte maturation.^10^ However, current studies view exosomes as a new and important paradigm in mediating intercellular communication, thus affecting the occurrence, development, and treatment of disease.^11^-^13^ In summary, exosomes are emerging as valuable sources of disease stage-specific information and indicators of disease progression. Similarly, exosomes have potential as biomarkers for diverse pathophysiological states and as therapeutic targets for complex human diseases.^14^, ^15^

In this review, we discuss the current research progress on exosomes, particularly regarding their role as intracellular couriers, biomarkers, and therapeutic vectors for pancreatic diseases. We also discuss shortcomings and issues among current studies that need further research. Finally, we discuss directions worthy of future research and applications of exosomes in pancreatic disease.

## 2. Exosomes: biogenesis, content, and function

Exosomes are nanosized, lipid bilayer membrane-enclosed extracellular vesicles (EVs) of endocytic origin.^16^ Via transmission electron microscopy (TEM), exosomes are seen to have classical 'cup' shapes with diameters of 30-150 nm.^17^-^19^ EVs are derived from intraluminal vesicles (ILVs) contained in multivesicular bodies (MVBs) within the endocytic system.^20^ After MVB docking and fusion with the plasma membrane, exosomes are secreted from the original cells into the extracellular milieu.^21^, ^22^ Currently, endosomal sorting complexes required for transport (ESCRT) are widely believed to play a regulatory role from exosome formation to secretion. In addition, Rab guanosine triphosphatase (GTPase) family members, such as Rab 11 and Rab 27, are important regulators linked to MVB trafficking and exosome secretion.^23^

Exosomes contain conserved proteins, such as tetraspanins (CD9, CD63, CD81), annexins and flotillin, heat shock proteins (HSP70 and HSP90), MHC class II-associated proteins, alix and tumor susceptibility gene 101 (TSG101) and other cell- or tissue type-specific proteins that reflect their cellular source of the exosomes.^24^ Exosomes have been widely demonstrated to carry mRNA and noncoding RNA (for example, microRNA (miRNA)), which can be transferred between cells and affect translation progression and downstream protein expression in recipient cells.^25^, ^26^ In addition to RNA, single-stranded DNA, genomic DNA, complementary DNA (cDNA), and transposable elements are contained in tumor-generated microvesicles.^27^

Recently, most studies view internalization as the primary method for exosome uptake; upon internalization, target cells can respond to the transferred exosomal cargo, regulating their basal function and gene expression.^28^ Exosomes have been widely studied in human disease. For instance, exosomes can be new crucial biomarkers in liquid biopsy, can participate in almost all aspects of the disease development process, and can even be engineered as 'drug carriers' for targeted therapy.^29^-^31^ Our discussion in the next section will focus on the research progress on and application of exosomes in pancreatic disease.

## 3. Exosomes and AP

**Figure 1:**
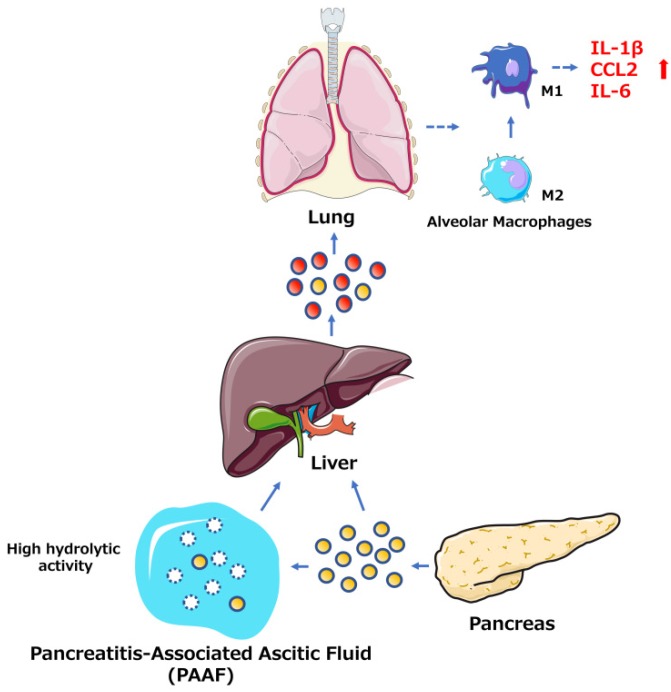
The role of exosomes in AP-related lung injury. The figure shows that the pancreas can release exosomes during AP (yellow circles, solid lines). Some exosomes directly reach the liver via the portal system, but most are largely retained in hepatic tissue. Another subset of exosomes released into PAAF are degraded by the hydrolytic activity of PAAF (white circles, dotted lines) and finally return to the hepatic tissue. In addition, the liver possibly generates and releases new exosomes during AP (red circles, solid lines). The exosomes can reach the alveolar compartment and transform alveolar macrophages into a proinflammatory phenotype. Moreover, AP circulating exosomes can markedly increase the expression of the proinflammatory cytokines IL-1β and IL-6 and the chemokine CCL2.

Among the many complications of AP, pulmonary dysfunction is the earliest and most important, responsible for up to 60% of all deaths occurring during the first week. Lipid derivatives, numerous proinflammatory cytokines, proteolytic enzymes and reactive oxygen species have been proposed to produce systemic effects during AP-related pulmonary failure; however, the mechanism related to the pancreatic damage is unclear.^32^, ^33^ Bonjoch et al. first clarified that exosomes are involved in AP, determining that during AP, the level of circulating exosomes is significantly increased and that these exosomes can penetrate the alveolar endothelial barrier to be engulfed by macrophages (Fig. 1). Moreover, exosomes activate alveolar macrophages by changing the macrophage phenotype from M2 to M1, causing lung injury in AP. In addition, proteomic analysis indicated that exosomes may originate from liver and immune cells, and exosomes in pancreatitis-associated ascitic fluid (PAAF) can be retained by the liver and absorbed by the portal system. These findings demonstrate that multiple organs are involved in generating circulating exosomes during AP and circulating exosomes may play a role similar to that of inflammatory factors in mediating a systemic cascade of inflammatory responses.^34^

Regarding molecular mechanisms, Zhao et al. used a microarray to examine exosomal miRNAs isolated from the culture medium of rat pancreatic acinar cells. The study identified 115 differentially expressed miRNAs (30 upregulated and 85 downregulated) and predicted the target gene expression of differentially expressed miRNAs connected with MAPK pathways.^35^ These findings are of considerable value for subsequent research on exosomal RNAs (exoRNAs) in AP.

Treatment for AP is primarily based on supportive therapy and includes pain relievers, antiemetics, fluid resuscitation, and oxygen administration.^36^ Moreover, clinicians lack effective therapies aimed at controlling proinflammatory mediators, which can be transferred via exosomes.^37^ Exosomes can protect their cargo from nucleases and proteases and thus may be promising therapeutic targets for systemic inflammation in AP.^38^

## 4. Exosomes and CP

CP predisposes patients to PC development through a common etiology: ductal metaplasia of acinar cells within the inflammatory environment of pancreatitis.^39^ Although only approximately 5% of CP patients will develop carcinoma over a period of 20 years, the relative risk for PC development continues to increase.^40^ Hence, the development of CP and transition from CP to PC deserves more extensive focus.

**Figure 2:**
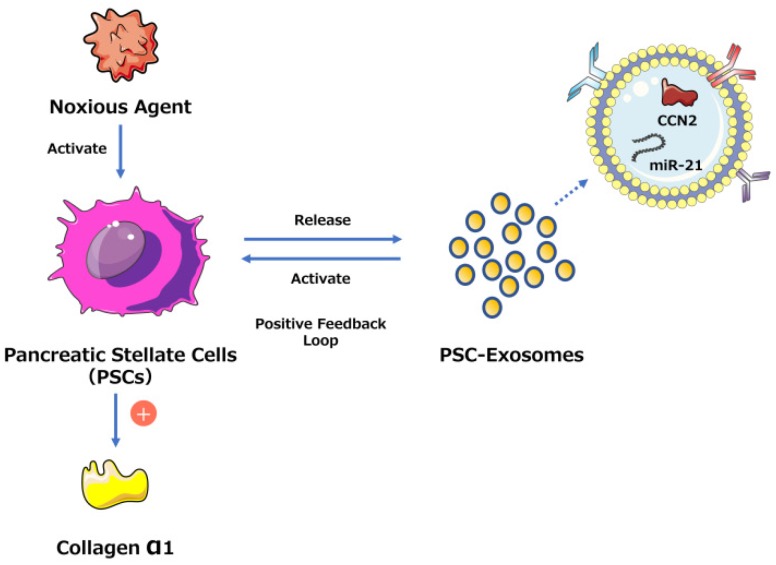
Exosome-mediated positive feedback loop during CP. The figure shows a positive feedback loop between PSCs and PSC-derived exosomes during CP. PSCs can release exosomes containing miR-21 and CCN-2, and these exosomes can activate PSCs to generate more exosomes and collagen α1. This loop can accelerate the development of pancreatic fibrosis during CP.

One study found that connective tissue growth factor (CCN2) upregulation in pancreatic stellate cells (PSCs) is closely related to increased miR-21 expression levels, which in turn stimulate CCN2 expression, indicating a positive feedback loop (Fig. 2). Moreover, both miR-21 and CCN2 have been identified in PSC-derived exosomes, which may mediate their delivery to PSCs, signifying that the exosomal miR-21-CCN2 axis is a novel pathway in PSC fibrogenic signaling.^41^

## 5. Exosomes and PC

### 5.1 Exosomes in molecular mechanisms of PC

#### 5.1.1. Exosomes in the interaction between PSCs and PC cells

**Figure 3:**
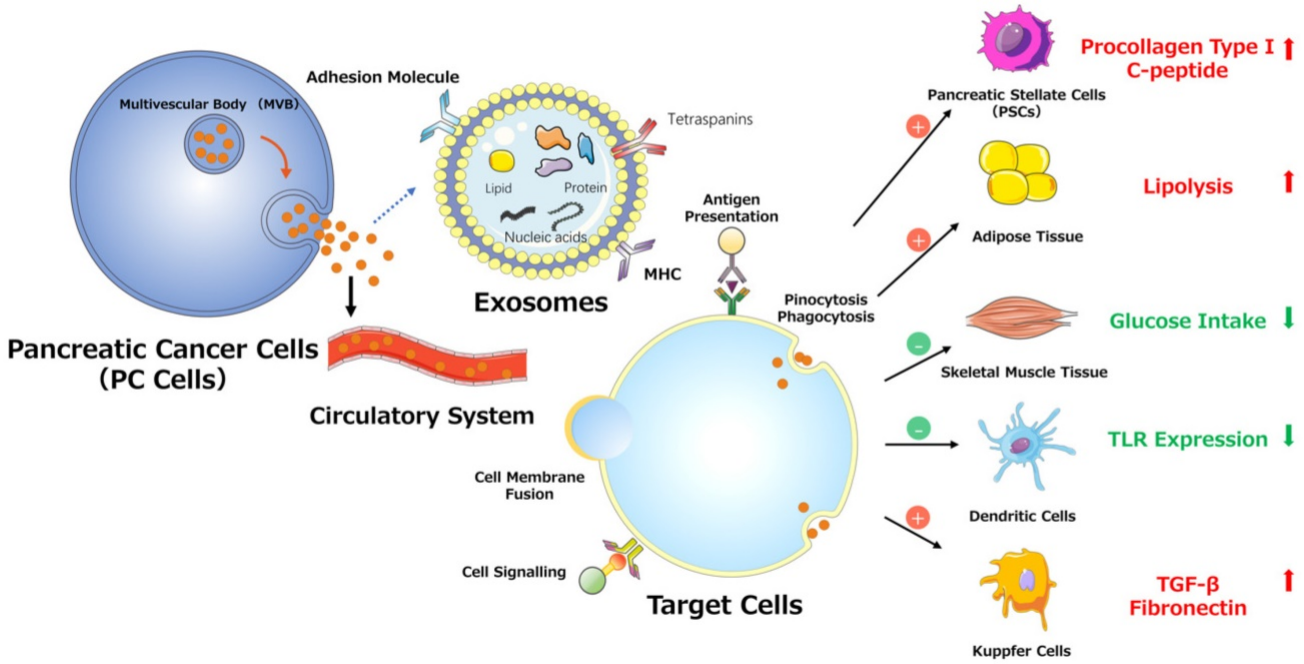
Various effects of exosomes on PC progression.

PSCs interact closely with cells, including endothelial, neuronal, and immune cells, and even cellular elements in the stroma. These interactions can facilitate the establishment of a suitable cancer microenvironment for pancreatic tumors.^42^ Recent studies indicate that miR-1246, miR-1290, and miR-21-5p are overexpressed in PSC-derived exosomes, which can be internalized by PC cells (PCCs). PSC-derived exosomes stimulate PC cell proliferation and migration and induce chemokine gene expression in PC cells. For example, the expression of several genes (CCL20, CXCL1, CXCL2, PDZK1IP1, SAA1, SAA2, SMCR7L, and ZNF619) is upregulated in both PANC-1 and SUIT-2 cells treated with PSC-derived exosomes.^43^ However, PC-derived exosomes can also affect PSCs. For example, PC-derived exosomes can promote the activation of Akt and ERK, enhance the mRNA expression of α-smooth muscle actin (ACTA2) and fibrosis-related genes, and increase the production of procollagen type I C-peptide in PSCs. Furthermore, miR-1246 and miR-1290 are overexpressed in PC-derived exosomes, and miR-1290 enhances the expression of ACTA2 and fibrosis-related genes in PSCs.^44^ These findings demonstrate that exosomes secreted by PC cells or PSCs play a unifying role in the pathogenesis and microenvironment of PC (Fig. 3).

#### 5.1.2. PC-associated diabetes mellitus

Chronic diabetes is considered an etiological factor for PC, as it modestly increases the risk for PC. In turn, new-onset diabetes, especially in the elderly, is likely a PC-associated complication and manifestation.^45^ Current data suggest that exosomes are involved in the process of PC-associated diabetes mellitus. Javeed et al. found that PC-derived exosomes contain adrenomedullin (AM) and carbohydrate antigen 19-9 (CA19-9), which inhibit insulin secretion by entering β-cells through caveolin-mediated endocytosis or micropinocytosis. In addition, paraneoplastic β-cell dysfunction could be caused by circulating PC-derived AM/CA19-9-positive exosomes, which inhibit insulin secretion through AM-induced endoplasmic reticulum (ER) stress and unfolded protein response (UPR) dysregulation.^46^ Glucose-dependent insulinotropic peptide (GIP) and glucagon-like peptide-1 (GLP-1) are incretins secreted by intestinal K and L cells.^47^ In one study, PC-derived exosomes inhibited insulin secretion by decreasing the levels of GIP and GLP-1 via suppressed expression of proprotein convertase subtilisin/kexin type 1/3 (PCSK1/3). Moreover, exosomal miRNAs (miR-6796-3p, miR-6763-5p, miR-4750-3p and miR-197-3p) have been identified and correlated with inhibitory effects on GIP and GLP-1 production.^48^

In addition to decreasing insulin secretion, tumors can induce glucose uptake/utilization dysregulation and insulin resistance (IR) in peripheral tissues, which is mediated by exosomes. PC-derived exosomes can trigger both the inhibition of glucose intake and lipidosis and can induce the translocation of glucose transporter 4 protein (Glut4) from the cell surface to the plasma membrane, which always permits facilitated diffusion of circulating glucose down the concentration gradient into muscle and fat cells. In addition, PC-derived exosomes can mediate IR in skeletal muscle cells through the insulin and PI3K/Akt/FoxO1 signaling pathways, and exosomal miRNAs may play pivotal roles in this process (Fig. 3).^49^

#### 5.1.3. Cancer-associated immune response

In communications between tumor and immune cells, exosomes perform a complex role in regulating tumor immunity via specific proteins and genetic components.^50^ As classical antigen-presenting cells (APCs), dendritic cells (DCs) express a variety of Toll-like receptors (TLRs).^51^ Evidence supports the involvement of TLRs (e.g., TLR2, TLR4, and TLR9) in PC development. 52 One study demonstrated that miR-203 is expressed in PC cells and exosomes and is significantly upregulated in DCs treated with PC-derived exosomes. Moreover, the TLR4, TNF-α, and IL-12 levels decrease after treatment with exosomes and miR-203 mimics but increase in PC-derived exosome-treated DCs via miR-203 inhibition. These results indicate that PC-derived exosomes may convert DCs into negative modulators to regulate the expression of TLRs in DCs via miR-203 (Fig. 3).^30^ Moreover, studies showed that high accumulation of TReg cells and minimal CD8+ T cell infiltration are observed in the tumor microenvironment in PDAC cells conditioned medium, mouse models and patients.^53^-^55^ As for the observed impaired infiltration of CD8+ T cells, Chen et al. offers a possible explanation. They found that melanoma cells-derived PD-L1-positive exosomes, could spread through the circulation and prevent the proliferation of CD8+ T cells as well as their infiltration in the tumor microenvironment. Therefore, if the same findings apply to PDAC, the results indicate that PDAC-derived exosomes containing PD-L1 may promote the impaired infiltration of CD8+ T cells in the tumor microenvironment.^56^, ^57^ Furthermore, recent studies demonstrated that the presence of M2 macrophages in the invasive front of PC contributed to PC progression, lymphangiogenesis and lymphatic metastasis, and correlated positively with poor survival.^55^, ^58^, ^59^ Linton et al. showed that PC-derived exosomes cause pro-tumor phenotype changes in macrophages. In addition, when macrophages were treated with the ascites-derived highly metastatic AsPC-1 PC cell line, they observed that the induction of this immunosuppressive phenotype in macrophages was more accentuated. Additionally, macrophages treated with AsPC-1-derived exosomes secrete increased amount of cytokines and growth factors, which promote PC progression, metastasis and angiogenesis.^60^

In addition to their inhibitory effect on the immune system, PC-derived exosomes have an active effect on the immune system in inhibiting PC progression. The deficiency of regulatory factor X-associated protein (RFXAP), a key transcription factor of the MHC II gene, can cause severe immunodeficiency via the inhibition of MHC class II expression and the inactivation of CD4+ T lymphocytes.^61^, ^62^ Ding et al. revealed that RFXAP is inhibited by miR-212-3p transferred from PC-derived exosomes, which decreases MHC II expression when released to DCs. Moreover, exosomal miRNAs can be transferred into DCs and inhibit target mRNA expression.^63^ Therefore, downregulation of miR-212-3p in PC cells or inhibition of the secretion of PC-derived exosomes might be explored as therapeutic strategies to prevent the inhibition of DC antigen-presenting function by miR-212-3p and promote the activation of anti-cancer immune responses.^57^, ^64^ The authors subsequently found that after depletion of exosomal miRNAs, PC-derived ultrafiltered exosome lysates (UELs) act as agonists, increasing immune activity via activating dendritic cells/cytokine-induced killer cells (DC/CIKs) to protect against PC progression. This effect may be mediated by exosomal proteins such as attractin; complement proteins C3, C4, and C5; integrin; and lactotransferrin; all of these are closely linked to lymphocyte activation, cell adhesion, immune regulation, or tumor inhibition.^65^, ^66^, ^67^

#### 5.1.4. Invasion and metastasis

Substantial evidence suggests that tumor-derived exosomes participate in and promote the formation of premetastatic niches, preparing a future metastatic site for the influx of tumor cells, engraftment, and the survival of incoming metastatic cells.^68^, ^69^, ^70^ Costa-Silva et al. revealed that pancreatic ductal adenocarcinoma (PDAC)-derived exosomes can be internalized by Kupffer cells, causing the secretion of transforming growth factor β and upregulation of fibronectin production by hepatic stellate cells (HSCs). These findings indicate that PDAC-derived exosomes can activate fibrotic pathways and the proinflammatory milieu to facilitate tumor cell metastasis. Moreover, these researchers revealed that macrophage migration inhibitory factor (MIF) was highly expressed in PDAC-derived exosomes, which likely primes the liver for metastasis and has prognostic and therapeutic significance (Fig. 3).^71^

In another study, researchers incubated PKH67-labeled highly metastatic Panc02-H7 cell-derived exosomes with Panc02 cells, which are weakly metastatic, and observed via fluorescence microscopy that exosomes are taken up by Panc02 cells. Furthermore, Panc02-H7 cell-derived exosomes increased the invasive and migratory capacities of Panc02 cells, as well as decreased Panc02 cell adhesion, which was mediated by the stromal cell-derived factor-1α receptor and downstream (CXCR4 and MMP-9) signaling pathways.^72^

During the progression of PC mediated by PC-derived exosomes, exosomal noncoding RNA is instrumental in tumor invasion and metastasis. Li et al. found that lncRNA Sox2ot isolated from exosomes of highly invasive PDAC cells promoted epithelial-mesenchymal transition (EMT) and stemness by acting as a competing endogenous RNA (ceRNA). Furthermore, lncRNA Sox2ot was overexpressed and was correlated with the tumor-node-metastasis (TNM) stage and overall survival rate in PDAC patients, and its levels decreased after tumor resection.^73^ In addition to identifying this lncRNA, this group identified a role of circRNAs in PC development, showing that high expression of a circRNA (circ-PDE8A) is correlated with lymphatic invasion, advanced TNM stage, and a poor survival rate in PDAC patients. Further study demonstrated that circ-PDE8A promotes the invasive growth of PDAC cells via the miR-338/MACC1/MET or AKT pathways.^74^ Recently, this research group showed that another circRNA (circ-IARS) could enter human microvascular vein endothelial cells (HUVECs) through exosomes and promote PC invasion and metastasis followed by increased endothelial monolayer permeability. Circ-IARS expression is positively correlated with liver metastasis, TNM stage and vascular invasion but negatively correlated with postoperative survival time.^75^

### 5.2. Diagnosis and prognosis

Diagnosis of patients with PC always happens upon presentation with recognizable clinical symptoms, necessitating subsequent blood and imaging tests. Currently, the efficacy of early PC detection by imaging techniques, such as computed tomography (CT) and endoscopic ultrasound (EUS), is unsatisfactory for disease prognosis and outcome.^76^, ^77^ Regarding serum biomarkers, CA19-9 has been generally applied for routine use in PC diagnosis, with sensitivity and specificity rates of nearly 85%, but confirming a diagnosis of PC using CA19-9 alone is difficult, particularly in patients presenting with nonspecific symptoms.^78^, ^79^ Thus, clinicians need more accurate and efficient indicators to aid in diagnosing PC, especially in early disease stages.

Compared with traditional techniques, exosome-based testing has particular properties and advantages: it is noninvasive (exosomes are available in multiple body fluids); exosomes are secreted at higher levels by tumor cells than by normal cells; many high-concentration biomarkers exist among cargo, allowing convenient isolation and analysis; and the cellular origin can be more accurately determined for exosomal biomarkers than for other circulating biomarkers.^80^-^83^ Although more rigorous clinical studies are needed for further validation, exosomes are gradually becoming an essential element of liquid biopsy.^84^ Here, we elaborate on the role of using exosomes in the diagnosis and prognosis of PC from the following three aspects: RNA, DNA, and proteins/protein compounds.(Table 1)

#### 5.2.1. miRNA

Exosomes carry mRNA and miRNA, acting as a shuttle for intercellular RNA transfer, and protect these molecules from RNase-dependent degradation, ensuring stable detection of RNA in circulating fluids.^84^ Therefore, the exploration of exosomal miRNAs as diagnostic biomarkers deserves further attention.

By RT-PCR analysis of PC patient serum miRNA, researchers found that miR-17-5p and miR-21 were overexpressed, with diagnostic sensitivities and specificities of 72.7% and 92.6% for miR-17-5p and 95.5% and 81.5% for miR-21.^65^ However, miR-21 is also significantly overexpressed in patients with other malignant tumors, including gastric, breast, ovarian, colon, and hepatic cancers, introducing doubt concerning its diagnostic value for discriminating PC from other tumors.^85^ ^86^ ^87^ ^88^ Exosomal miR-10b, miR-20a, miR-21, miR-30c, miR-106b, and miR-181a were overexpressed in PDAC plasma from clinical samples, while exosomal miR-let7a and miR-122 exhibited low expression. Moreover, the elevated levels of exosomal miR-10b, miR-20a, miR-21, miR-30c, miR-106b and the reduced level of miR-let7a normalized after tumor resection. Furthermore, the sensitivity and specificity of the abovementioned exosomal miRNAs were nearly 100% for discriminating the PDAC group from the CP and healthy groups.^89^ Madhaven et al. employed flow cytometry to examine selected PC-initiating cells (PaCICs) markers (CD44v6, Tspan8, EpCAM, MET, and CD104) in exosomes from patient serum and used qRT-PCR to measure miRNA levels (miR-1246, miR-4644, miR-3976, and miR-4306) in serum exosomes and exosome-depleted serum. Upon PC diagnosis, the sensitivity/specificity of PaCIC markers, miRNAs, and the combination of both were 0.96/0.86, 0.81/0.94 and 1.00/0.80, respectively.^90^

In addition to examining different miRNAs, some researchers have attempted to enhance the efficiency of exoRNAs for diagnosis through technological improvements. An ultrasensitive localized surface plasmon resonance (LSPR)-based miRNA sensor with single-nucleotide specificity was developed using chemically synthesized gold nanoprisms attached to a solid substrate with unprecedented long-term stability and reversibility. Researchers applied this sensor to identify levels of exosomal miRNA-10b in PC cell culture media and human plasma and showed that miRNA-10b is significantly overexpressed in PDAC plasma-derived exosomes.^91^ Taller et al. introduced a novel technique for exoRNA diagnosis called on-chip surface acoustic wave (SAW) lysis and ion-exchange nanomembrane detection. This work presented a microfluidics-based approach for exoRNA analysis based on SAW exosome lysis and ion-exchange nanomembrane RNA sensing performed in conjunction on two separate chips. Upon detection of the model target has-miR-550 in PC cell media, the SAW-based exosome lysis rate was 38%. Compared to traditional exoRNA detection techniques, this platform exhibits advantages for PC diagnosis, such as decreased time and sample volumes and minimal sample loss.^92^ Recently, Ko et al. developed the exosome sorting track-etched magnetic nanopore (ExoTENPO) to promote the efficacy of differentiating cancer and precancer patients from healthy controls. This group applied a machine learning algorithm to produce predictive panels to accurately evaluate and identify samples from heterogeneous cancer-bearing individuals. By analyzing linear combinations of eight mRNA profiles per panel from 34 clinical samples obtained from patients with untreated metastatic PC and healthy controls, this technique classified every patient into the correct group. Recently, this group also identified a biomarker panel of 11 EV miRNAs to effectively distinguish PDAC mice from healthy mice or mice with precancerous lesions.^93^, ^94^

#### 5.2.2. DNA mutations

Exosomes contain >10 kb of double-stranded genomic DNA fragments, and mutations in KRAS and p53 can be detected by analysis of PC-associated exosomal genomic DNA, indicating that exosomes can aid in identifying genomic mutations in patients with PC.^95^ Lucas et al. performed comprehensive profiling of exosomal DNA (exoDNA) and exoRNA by whole genome, exome, and transcriptome sequencing and determined that multiple actionable mutations, including alterations in NOTCH1 and BRCA2, can be identified in exoDNA sequencing data and that fusion genes related to tumor neoantigens can be detected in exoRNA sequencing data.^96^ Yang et al. conducted a proof-of-concept study to explore the clinical utility of circulating exoDNA for the identification of KRAS^G12D^ and TP53^R273H^ mutations in patients and healthy controls. The results highlight circulating exoDNA as a rapid and low-cost diagnostic marker to identify PC-driving mutations. However, mutations can be detected both in patients with intraductal papillary mucosal neoplasms (IPMNs) and in healthy subjects, suggesting that exoDNA biopsy is more suitable for the assessment of cancer risk than for definitive cancer diagnosis.^97^ Similar to exoDNA, circulating cell-free tumor DNA (cfDNA) can be used to detect KRAS mutations in many gastrointestinal tumors.^98^ Allenson et al. compared the diagnostic value of exoDNA and cfDNA for identifying PDAC patients via KRAS mutations. ExoDNA was identified in 7.4%, 66.7%, 80%, and 85% of age-matched controls and patients with localized, locally advanced, and metastatic PDAC, respectively, while KRAS cfDNA was detected in 14.8%, 45.5%, 30.8%, and 57.9% of these same groups, suggesting that exoDNA is more valuable than cfDNA for PDAC diagnosis.^99^

#### 5.2.3. Proteins/protein compounds

Glypicans (GPCs) comprise a family of heparin sulfate proteoglycans (HSPGs), which attach to the exocytoplasmic domain of the cell membrane by a glycosylphosphatidylinositol (GPI) anchor.^100^ ^101^ GPC1 is overexpressed in human PC, and expression of the antisense sequence can apparently decrease the tumorigenicity of PC cells.^102^ A study by Melo et al. reported that high levels of GPC1+ circulating exosomes (crExos) were found in serum from 190 PDAC patients relative to the levels in healthy donors (P<0.0001) and that GPC1+ crExos contained oncogenic KRAS^G12D^. Importantly, GPC1+ crExos presented 100% sensitivity and specificity in discriminating patients with almost every stage of PC from those with benign pancreatic disease (BPD) and healthy controls. 95 103 However, whether GPC1+ crExos can diagnose PC as efficiently as the paper suggests remains controversial. On the one hand, the PDAC patients selected in the study included those with all stages of PC, and most cases were unresectable and incurable. Thus, diagnosis of PDAC patients via this biomarker may lead to a low rate of early resection and poor long-term survival. Biospecimen collection should be prioritized before resection for patients with stage I disease.^103^ On the other hand, a recent study found that GPC1+ crExos are also overexpressed in stage III colon cancer, indicating that GPC1+ crExos are not a specific marker for diagnosing PC.^104^ In addition, some researchers believe that, circulating exosomal miRNAs, such as miR-10b and miR-20a, are more specific and accurate for PC diagnosis than GPC1+ crExos.^89^

Zinc transporter protein 4 (ZIP4), a membrane-localized zinc ion transporter regulating intracellular zinc homeostasis, was proven to be differentially expressed in multiple cancers and to be related to the progression of cancers, including PC.^105^, ^106^ Via proteomic analysis, Tan et al. identified ZIP4 as the most highly upregulated exosomal protein in PC-1.0 (a highly malignant PC cell line) cells and demonstrated that exosomal ZIP4 can significantly promote PC growth *in vivo* and *in vitro*. Moreover, the level of serum exosomal ZIP4 was appreciably higher in samples from the malignant PC group (n=24) than in those from the benign pancreatic disease group (n=32, P<0.0001), biliary disease group (n=32, P=0.0053) or healthy group (n=46, P<0.0001), showing promising diagnostic efficacy for PC.^107^

In addition to GPC1+ crExos and ZIP4, which have statistically proven value, many other exosomal proteins have potential as biomarkers for PC diagnosis. A disintegrin and metalloprotease (ADAM) 10 and 17 are largely responsible for the generation of soluble MHC class I (MHCI)-related chain molecules A and B (MICA/B), which are correlated with tumor progression.^108^ Another study demonstrated a tumor cell-specific role of ADAM10 and/or ADAM17 in the shedding MICA and/or MICB and found that exosomal ADAM10 and ADAM17 shedding of MHCI has potential for PC diagnosis.^109^ Epidermal growth factor receptor (EGFR) participates in the progression of PDAC, especially in invasion and the acquisition of aggressive clinical behaviors.^110^ EGFR and its ligands, EGF and TGFα, are overexpressed in serum in most cases of PC.^111^ A recent study showed that PC cells secrete a soluble form of EGFR (sEGFR) into exosomes, presumably by ectodomain shedding,^112^ indicating that exosomal sEGFR may help diagnose PC and track the therapeutic response.^113^ Furthermore, MIF, a proinflammatory cytokine and an important regulator of the innate immune response,^114^ ^115^ is overexpressed in PDAC-associated exosomes, and liver premetastatic niche formation and metastasis can be inhibited by blocking exosomal MIF, indicating the potential of this cytokine in the evaluation of PC prognosis.^71^

In addition to conventional methodological approaches, some new methods for detecting exosomal proteins are equally worthy of attention. Recently, a study showed that tumor exosomes can activate transcription in saliva gland cells, altering the proteomic and transcriptomic profiles of saliva gland cell-derived exosomes.^116^ Further study revealed that discriminatory biomarkers can be identified in the saliva of PC-engineered C57BL/6 mouse models, revealing a promising, noninvasive and easily accessible detection method using specific exosomal transcriptomic biomarkers in saliva.^117^ Kong et al. developed effective and simple polydopamine-modified immunocapture substrates and an ultrathin polydopamine-encapsulated antibody-reporter-Ag(shell)-Au(core) multilayer (PEARL) surface-enhanced Raman scattering (SERS) nanotag with the quantitative signal that achieved ultrasensitive and specific detection of PC-derived exosomes. Moreover, these researchers reported that the MIF antibody-based SERS immunoassay not only can discriminate PC patients from healthy controls but also can distinguish metastasized tumors from metastasis-free tumors and TNM P1-2 stage tumors from P3 stage tumors (with a sensitivity of 95.7%). Therefore, this technique based on an exosomal protein immunoassay provides an effective tool for the early detection, classification and metastasis monitoring of PC.^118^

### 5.3. Treatment

Because of the drug loading and signal carrying capacity of exosomes, their potential use in drug delivery and therapy has recently received much attention.^119^ Currently, research on exosomal drug delivery for PC treatment has mainly focused on loading genetic substances—for example, small interfering RNA (siRNA) and miRNA—into exosomes to inhibit PC progression and metastasis. Recent advances in gene therapies offer novel opportunities for treatment in addition to aggressive chemotherapy and surgical resection, even in patients with locally advanced disease.^120^ KRAS mutations are demonstrated to occur early in the development of PC, consistently manifesting as a gain-of-function substitution mutation in codon 12 that mutates the glycine residue to aspartate (G12D).^121^ ^122^ By loading siRNA and short hairpin RNA (shRNA) targeting KRAS^G12D^ into exosomes (called iExosomes), Kamerker et al. observed that iExosomes markedly decreased the levels of the phosphorylated ERK protein (a major mediator of KRAS activation) and KRAS^G12D^ mRNA in human PANC-1 cells. In a mouse model, pancreatic tumor growth and metastasis formation were significantly suppressed after peritoneal injection of iExosomes. Subsequent tumor histopathology results also suggested improvements in tumor pathology. Moreover, during this process, CD47 on exosomes conferred protection against circulating monocyte-dependent phagocytosis, enhancing the therapeutic efficacy of iExosomes.^123^ Shortly thereafter, Mendt et al. developed a large-scale, bioreactor-based method of production method for clinical-grade exosomes to meet the good manufacturing practice (GMP) standard. The exosomes are generated from bone marrow-derived MSCs and electroporated with siRNA targeting PDAC Kras^G12D^ using a clinical-grade diluent (Plasma-Lyte). *In vivo*, the exosomes suppressed the growth of highly metastatic, patient-derived PDAC xenografts in mice, increasing survival in PC mouse models and indicating a similar good antitumor effect and stability with no measured side effects.^124^

Substantial evidence indicates that Smad3, an intracellular direct mediator of the TGF-β signaling pathway, plays an essential role in TGF-β-mediated EMT during PDAC proliferation and metastasis. 125 126 Li et al. successfully loaded exogenous miR-145-5p into exosomes from human umbilical cord mesenchymal stromal cells (hucMSCs), which was proven to be safe for use in animal models and exhibited intrinsic therapeutic effects in hepatic and heart disease. In vitro, these exosomes inhibited PDAC cell proliferation and invasion and increased apoptosis and cell cycle arrest, followed by decreased Smad3 expression. Furthermore, they significantly reduced xenograft tumor growth *in vivo*. These findings provide novel insight suggesting that exosomes may be an attractive therapeutic vehicle for the clinical administration of miRNAs in PDAC patients.^127^

In addition to functioning as a drug delivery system, exosomes can be used directly as therapeutic agents for PC. Currently, gemcitabine (GEM) is usually the recommended first-line chemotherapeutic agent for PC and is administered alone or in combination with other agents.^128^ However, gemcitabine sometimes has only limited efficacy in extending patient survival, likely due to innate or acquired chemoresistance mechanisms.^129^ Overcoming drug resistance during chemotherapy is challenging. Studies demonstrate that exosomes can regulate chemoresistance in cancer, enhancing drug resistance in cancer cells by directing drug export, transporting drug efflux pumps, and exchanging miRNAs among cells.^130^ Richards et al. reported that treatment of cancer-associated fibroblasts (CAFs) with gemcitabine significantly enhanced the survival and proliferation of PC cells. Furthermore, exosomes (EVs) secreted from GEM-treated CAFs increased the expression of Snail (a promoter-binding transcription factor), possibly via miR-146a.^131^ In further mechanistic studies, researchers demonstrated that in PDAC cells, compared to gemcitabine alone, survivin-containing exosomes significantly increased the effect of apoptotic cell death.^132^ Furthermore, gene expression analyses of gemcitabine chemoresistance-related exosomes (Gem-Exos) showed downregulation of DCK (a gemcitabine-metabolizing gene) and upregulation of SOD2 and CAT (ROS-detoxifying genes). These findings suggest that SOD/CAT suppress basal and gemcitabine-induced ROS production by exosome-mediated transfer of their transcripts and that DCK downregulation may be induced by exosome-delivered miR-155.^133^

Additionally, further researches on reprogramming of PC-derived exosomes have suggested that expression of superantigens to activate T cells could promote immune responses in the PC tumor environment. As a powerful superantigen, staphylococcal enterotoxin (SEB) has shown its ability to stimulate not only T cell proliferation and activation but the Fas-mediated apoptotic pathway.^134^ Mahmoodzadeh Hosseini et al. demonstrated that hybrids of MIA PaCa-2 exosomes and SEB (EXO/SEB) promote anti-proliferative effects and cell death in PC cells.^135^ The results highlight the potential of reprogramming of exosomes as a therapy in PC treatment.

## 6. Issues and prospects

The isolation and identification of exosomes is the first and most crucial step in all exosome studies to date. Currently, differential ultracentrifugation is regarded as the most effective technique to obtain relatively pure exosomes from samples; however, no current isolation or purification technique can separate exosomes with 100% purity.^136^ Thus, exosomes in most studies concerning pancreatic disease actually represent mixed EV populations, and an urgent problem to be solved is how to compare the different subtypes of EVs to determine their potential specific or prominent functions.^137^ Past studies have shown exosomes to be a “double-edged sword”, not only promoting cancer proliferation but also suppressing tumor progression.^30^, ^71^ ^133^ Therefore, classifying exosome subgroups according to their functions and mechanisms is an equally important goal. Recently, Zhang et al. classified nanoparticle components of the cellular communication milieu according to particle size via asymmetric flow field fractionation (AF4). Based on the presence of at least two previously reported exosome subgroups, researchers classified small and large exosomes (Exo-S and Exo-L, respectively) and identified a formerly unrecognized nanoparticle called an exomere. Notably, these three nanosized particles exhibited diverse lipophilic, proteomic, DNA and RNA profiles and N-glycosylation patterns, suggesting that they originate via different biogenesis mechanisms.^138^ ^139^ Undoubtedly, this finding represents significant progress in understanding the role of various exosome subtypes in diverse intractable conditions, such as pancreatic diseases. In past studies on pancreatic disease and exosomes in human or animal models, researchers focused on exosomes isolated from biofluids, such as circulating blood, urine, and cerebrospinal fluid. Exosomes in biofluids may in fact derive from multiple organs, and developing approaches to distinguish these exosomes and determine their organ of origination remains a difficult problem. Recently, Vella et al. introduced a rigorous approach for isolating exosomes from brain tissue. Using a novel method, these researchers successfully enriched and characterized exosomes from the human frontal cortex. More importantly, exosomes also maintain their vesicle and cargo integrity via their endosome-derived origin.^140^ This novel method will provide significant value in isolating exosomes from the pancreas and facilitate more detailed insight into pancreatic exosomes.

In the diagnosis of AP, exosomes have potential as biomarkers for AP and its complications. For instance, miR-127 levels are significantly positively correlated with histopathological severity scores of the pancreas and lungs in AP, and the levels are increased in AP with lung injury.^141^ Serum levels of protein carbonyl groups begin to rise early in the course of ischemia-reperfusion AP and decrease at later stages, suggesting that this factor could be an effective biomarker for the diagnosis of early stages of AP.^142^ Therefore, determining whether the combination of exosomes and miRNA or protein can enhance the diagnostic value of serum biomarkers for AP is worthy of further study. In the terms of mechanisms, studies on necroptosis in AP demonstrated acinar cell necroptosis and its potential value for regulating inflammatory injury.^143^ Moreover, one study demonstrated that miR-21 promotes regulated necrosis involving RIP3-dependent regulated necrosis (necroptosis) and that miR-21 inhibition effectively reduces the severity of AP.^144^ Considering that exosomes carry miRNAs, including miR-21, exosomes may also participate in the process of necroptosis in AP, a possibility deserving more detailed examination.^145^ ^146^

The diagnosis of CP is always obvious in advanced cases, but diagnosis in early stages is challenging.^3^ The findings show that the miRNA expression profile is different between early and late CP. Among the identified miRNAs, has-miR-221 and has-miR-130a are biomarkers of early CP, and a panel of serum miRNAs has potential for clinical application in the early diagnosis of CP.^147^ In terms of CP-to-PC conversion, Mayerle et al. demonstrated that compared to CA19-9 alone, a biomarker signature (nine metabolites and CA19-9) improved the diagnosis of PDAC from CP and treatment stratification.^148^ Therefore, these findings deserve additional studies to determine whether these molecules can be found in exosomes, and according to their stability and targeting, whether exosomes can further improve the diagnosis of and therapy for CP.

In studies of treatment of PC, compared to classical antitumor drugs and lipid carriers or liposomes, exosomes have the advantage of good tumor targeting ability. However, in two studies about exosomal targeting of oncogenic KRAS in PC, published by Kamerker et al. and Mendt et al., after injection of exosomes into mouse models, a large number of exosomes were aggregated and obtained from both the liver and spleen, in addition to the pancreas.^123^ ^124^ Hence, there is still much room for improvement in exosomal targeting in the therapeutic setting, and future studies will focus on potential side effects on the liver and spleen. In addition, many other urgent problems also need resolution, such as increasing the purity and productivity of cargo-loaded exosomes, determining and controlling the dose of exosomes in clinical trials, and—more importantly—performing repeated testing for potential side effects when used in humans. However, the discovery of cargo-loaded exosomes marks an essential step forward on the road to the clinical application of exosomes and presents an important strategy for other oncogenes and tumors.

In studies of PC biomarkers based on exosomal miRNAs, results may differ due to the sampling times throughout the day. The reason may be that the RNA life cycle is generally confirmed to be regulated in a circadian manner, contributing to circadian gene expression. Studies found that the production and degradation of RNA by miRNA might maintain the circadian pattern and rhythm.^149^ In addition to possible influences due to circadian rhythms, although miRNA has organ and tissue specificity, the same miRNA or other noncoding RNA can be derived from multiple organs and tissues.^89^ ^150^ These observations undoubtedly dampen the prospects for the future application of miRNAs in the early diagnosis of PC and other diseases. 73 Actually, exosomal lncRNAs/circRNAs also exhibit good stability and organ/tissue specificity, and their diagnostic value as biomarkers has been demonstrated in other gastrointestinal tumors.^151^ ^152^. Other challenges in the use of exosomes for clinical diagnosis are process portability and actual cost. Recently, Lewis et al. demonstrated a novel and simple approach for integrating the capture and analysis of EVs, including exosomes, directly from serum, plasma, or whole blood onto an AC electrokinetic microarray chip. This initial study validated the good diagnostic value of this method for detecting PDAC through the presence of glypican-1 and CD63.^153^ The method, dependent on the ACE chip, integrates the traditional complex processes of sample preparation, exosome isolation and identification, and device or instrument analysis into a simple and effective sampling and analysis model.

## 7. Conclusion

In summary, as a complex and dangerous clinical condition, pancreatic disease has always presented difficulty for clinicians and researchers to overcome. We lack efficient treatment for the early stages of AP due to uncertainties concerning its etiology. The primary therapy for CP is treatment of complications, and monitoring the transition from CP to PC is difficult. The current techniques for the early detection of PC, including serum biomarkers, imaging modalities, and pathological biopsy, seem ineffective at enhancing the survival rates of PC patients. As novel mediators of cellular communication, exosomes participate in all steps of pancreatic disease and have clear potential as treatment targets in pancreatic disease. However, we must remember to be cautious and consider the potential problems of using exosomes, especially the safety, dose-response, and side effects. The study of exosomes is still in its infancy, and additional extensive research is required before future clinical application.

## Figures and Tables

**Table 1:** Reported exosomal PC biomarkers

Exosomal Biomarkers	Exosome Isolation Method	Sample	Sensitivity Specificity	Efficacy	Ref.
miR-17-5p miR-21	Ultracentrifugation	Human serum	72.7%; 92.6% 95.5%; 81.5%	Discriminating PC from non-PC and healthy individuals	65
miR-10b miR-21 miR-30c	Ultracentrifugation	Human plasma	100%; 100% 100%; 100% 100%; 100%	Superior to exosomal GPC1 or CA19-9 in diagnosis of PDAC and differentiating between PDAC and CP	86
Combination miRs and and PC-initiating cells markers	Sucros-gradient centrifugation	Human serum	100%; 80%	Allowing for a highly sensitive and minimally invasive PC diagnostics	90
hsa-miR-550	Surface acoustic wave (SAW)-driven exosomes lysis	PC cell media	Not tested	Time saving, smaller sample volume needed, and minimal sample loss for PC diagnosis.	92
miR-10b (by ultrasensitive localized surface plasmon resonance (LSPR)-based microRNA sensor)	Sequential Ultracentrifugations	PC cell media	Not tested	Novel diagnostic strategies for PC based on direct quantitative measurement of plasma and exosome microRNAs	91
miRNAs (a panel of 11 miRNAs)	Exosomes track-etched magnetic nanopore (TENPO)	Mice plasma	Not tested	Distinguishing mice with PDAC from either healthy mice or thoses with pre-cancerous lesions	93,94
exoDNA exoRNA	Ultracentrifugation	Human plasma and pleural effusion	Not tested	Detecting alterations in NOTCH1 and BRCA2 in exoDNA data and PC neoantigens-related fusion genes in exoRNA data	96
exoDNA (KRAS^G12D^ and TP53^R273H^ mutations)	Ultracentrifugation	Human serum	Not tested	More suitable for assessment of PC risk	97
Proteoglycan Glypican-1	Ultracentrifugation	Human serum	100%; 100%	Discriminating almost each stage of PC (carcinoma in situ, stage I as well as stages II-IV) from BPD and healthy controls	103
Zinc transporter protein 4 (ZIP4)	SBI ExoQuick-TC kit	Human serum	Not tested	Obviously higher level in PC group than BPD group and biliary disease group and healthy group	107
A disintegrin and metalloproteases (ADAM) 10 and 17	SBI ExoQuick-TC kit	PC cell media	Not tested	Potential for PC diagnosis	109
Soluble epidermal growth factor receptor (sEGFR)	Ultracentrifugation	PC cell media	Not tested	Indicative of PC diagnosis and tracking response to therapy.	112,113
Macrophage migration inhibitory factor (MIF)	Ultracentrifugation	PC cell media	Not tested	Predicting metastasis and prognosis of PDAC	71
MIF	The PDA chip and PEARL SERS Tag-based exosomes sensors	PC cell media	Not tested	Distinguishing metastatic from non-metastasis PC, and P1-2 stages from P3 stage PC, without the need of histopathological examination	118

## Abbreviations

AP: Acute pancreatitis
CP: Chronic pancreatitis
PC: Pancreatic cancer
EV: Extracellular vesicles
TEM: Transmission electron microscopy
ILVs: Intraluminal vesicles
MVBs: Multivesicular bodies
GTPase: Guanosine triphosphatase
HSP: Heat shock protein
TSG101: Tumor susceptibility gene 101
miRNA: MicroRNA
lncRNA: Long noncoding RNA
circRNA: Circular RNA
cDNA: Complementary DNA
PAAF: Pancreatitis-associated ascitic fluid
exoRNAs: Exosomal RNAs
CCN2: Connective tissue growth factor 2
PSC: Pancreatic stellate cell
AM: Adrenomedullin
ER: Endoplasmic reticulum
GIP: Glucose-dependent insulinotropic peptide
GLP-1: Glucagon-like peptide-1
UPR: Unfolded protein response
PCSK1/3: Proprotein convertase subtilisin/kexin type 1/3
IR: Insulin resistance
Glut4: Glucose transporter 4 protein
APCs: Antigen-presenting cells
DCs: Dendritic cells
TLRs: Toll-like receptors
RFXAP: Regulatory factor X-associated protein
UEL: Ultrafiltered exosome lysates
CIKs: Cytokine-induced killer cells
PDAC: Pancreatic ductal adenocarcinoma
HSCs: Hepatic stellate cells
MIF: Migration inhibitory factor
EMT: Epithelial-mesenchymal transition
ceRNA: Competing endogenous RNA
TNM: Tumor-node-metastasis
HUVECs: Human microvascular vein endothelial cells
CT: Computed tomography
EUS: Endoscopic ultrasound
PaCICs: PC-initiating cells
LSPR: Localized surface plasmon resonance
SAW: Surface acoustic wave
ExoTENPO: Exosome sorting track-etched magnetic nanopore
exoDNA: Exosomal DNA
GPCs: Glypicans
HSPGs: Heparin sulfate proteoglycans
GPI: Glycosylphosphatidylinositol
BPD: Benign pancreatic disease
ZIP4: Zinc transporter protein 4
ADAM: A disintegrin and metalloprotease
EGFR: Epidermal growth factor receptor
PEARL: Polydopamine-encapsulated antibody-reporter-Ag(shell)-Au(core) multilayer
SERS: Surface-enhanced Raman scattering
siRNA: Small interfering RNA
shRNA: Short hairpin RNA
GMP: Good manufacturing practice
hucMSCs: Human umbilical cord mesenchymal stromal cells
GEM: Gemcitabine
CAFs: Cancer-associated fibroblasts
SEB: Staphylococcal enterotoxin
